# Cardiovascular adverse events induced by immune checkpoint inhibitors: A real world study from 2018 to 2022

**DOI:** 10.3389/fcvm.2022.969942

**Published:** 2022-08-10

**Authors:** Si Wu, Hansheng Bai, Ling Zhang, Jiamin He, Xiangru Luo, Shiyi Wang, Guangjun Fan, Na Sun

**Affiliations:** Department of Pharmacy, The Second Affiliated Hospital of Dalian Medical University, Dalian, China

**Keywords:** immune checkpoint inhibitors, cardiovascular adverse events, immunotherapy, predictors, surveillance factors

## Abstract

**Background:**

The reported rate of cardiovascular adverse events (CAE) caused by immune checkpoint inhibitors (ICI) is low but potentially fatal. Assess the risk of CAE in cancer patients and compare the incidence of CAE between Chinese developed ICIs and imported ICIs.

**Methods:**

A retrospective analysis was performed on cancer patients treated with ICI for at least four cycles in the Second Affiliated Hospital of Dalian Medical University from January 2018 to March 2022. Baseline characteristics, physiological and biochemical values, electrocardiographic and echocardiographic findings were compared between patients with and without CAE.

**Results:**

Among 495 patients treated with ICIs, CAEs occurred in 64 patients (12.93%). The median time to the event was 105 days (61–202). The patients with low neutrophil-to-lymphocyte ratio (L-NLR) were significantly associated with the risk of developing CAE (hazard ratio HR 3.64, 95% confidence ratio CI 1.86–7.15, *P* = 0.000). Patients with higher comorbidity burden significantly increased the risk of developing CAE (HR 1.30, 95% CI 1.05–1.61, *P* = 0.014). Those who received a combination of ICI and vascular endothelial growth factor receptor (VEGFR) inhibitors (HR 2.57, 95% CI 1.37–4.84, *P* = 0.003) or thoracic radiation therapy (HR 32.93, 95% CI 8.81–123.14, *P* = 0.000) were at a significantly increased risk of developing CAE. Compared to baseline values, creatine kinase is -oenzymes (CK-MB) (95% CI -9.73 to -2.20, *P* = 0.003) and cardiac troponin I (cTnI) (95% CI -1.06 to -0.06, *P* = 0.028) were elevated, and the QTc interval prolonged (95% CI -27.07 to -6.49, *P* = 0.002). Using nivolumab as a control, there was no difference in CAE risk among the eight ICIs investigated. However, the results of the propensity matching showed that programmed death-ligand 1 (PD-L1) inhibitors had lower CAE occurrence compared with programmed cell death protein 1 (PD-1) inhibitors (adjusted HR = 0.38, *P* = 0.045).

**Conclusion:**

Patients who received concurrent VEGFR inhibitors and ICIs had a history of thoracic radiation therapy, L-NLR, and higher comorbidity burden had an increased risk of CAEs. Elevated cTnI, CK-MB, and QTc, can be used to monitor CAEs. There was no significant difference in CAE risks between Chinese domestic and imported ICIs. PD-L1 inhibitors had lower CAE occurrence than PD-1 inhibitors.

## Introduction

Immunotherapy, such as immune checkpoint inhibitors (ICIs), has revolutionized cancer treatment in recent years. ICIs significantly improve the survival rate of patients with advanced cancer. ICIs are monoclonal antibodies against cytotoxic T lymphocyte-associated protein 4 (CTLA-4), programmed cell death protein 1 (PD-1), and programmed death-ligand 1 (PD-L1). These agents function by blocking an immune checkpoint at the coreceptor and ligand interface of the T-cell and the antigen-presenting cell (anti-CTLA-4) or by inhibiting the interaction between the T cell and the tumor cell (anti-PD-1 and anti-PD-L1), allowing the increased destruction of cancer cells (1, 2). They have unusual adverse reactions that are different from traditional chemotherapy drugs. With the widespread application of immunotherapy, ICIs can lead to a wide range of immune-related adverse events (irAE). ICIs can induce irAE in multiple organs due to non-specific immune system activation. Most irAEs are manageable in the early stage, but about 10–17% lead to fatal consequences (3). Among the adverse reactions caused by ICIs, the reported rate of cardiovascular adverse events (CAEs) is low, but the mortality rate is high, which may lead to irreversible consequences (4).

CAEs may pose potential physical and economic threats to patients. Most studies on ICI-related cardiotoxicity are case reports, and the incidence of CAEs can be underestimated. Furthermore, the relationship between CAEs and ICIs and the potential associated factors is unclear. Currently, there are eight ICIs being used in China: nivolumab (PD-1), pembrolizumab (PD-1), atezolizumab (PD-L1), sintilimab (PD-1), camrelizumab (PD-1), toripalimab (PD-1), tislelizumab (PD-1), and durvalumab (PD-L1). Among these ICIs, sintilimab, camrelizumab, toripalimab, and tislelizumab were developed in China. There is no comparative study of the effects of domestic ICIs and imported ICIs on CAEs. This study was designed to: (1) provide estimates of the incidence of ICI-related CAEs, (2) determine the clinical characteristics of patients associated with the risk of developing ICI-related CAEs, and (3) compare the differences in CAE between domestic and imported ICIs. This study did not include anti-CTLA-4 drugs due to their limited availability in China.

## Materials and methods

### Study population and data collection

The study was carried out at the Second Affiliated Hospital of Dalian Medical University. Inclusion criteria were patients 18 years or older who received at least four cycles of ICI treatment from January 2018 to March 2022. The patients were stratified according to whether CAE occurred within 1 year of ICI treatment. The exclusion criteria were patients with a history of severe cardiac disease or patients with incomplete clinical data. The study was approved by the institutional review board of the Second Affiliated Hospital of Dalian Medical University (approval number 2020 NO.044, approval date 2020-11-27).

The following data were collected: age, gender, comorbidities, tumor type, chemotherapies, radiation therapy, vascular endothelial growth factor receptor (VEGFR) inhibitors, human epidermal growth factor receptor 2 (HER-2) inhibitors, epidermal growth factor receptor-tyrosine kinase inhibitors (EGFR-TKI), PD-1 and PD L-1 inhibitors, PR interval, QTc interval, ejection fraction (EF), creatine kinase (CK), CK-MB, cardiac troponin I (cTnI), B-type natriuretic peptide (BNP), and the occurrence time and type of CAEs. The Charlson Comorbidity Index (CCI) score and the neutrophil-to-lymphocyte ratio (NLR) were calculated for all patients. NLR < 3 can be specified as L-NLR.

### Immune checkpoint inhibitors-related cardiovascular adverse events

ICI-related CAEs were defined as CAEs diagnosed within 1 year after the first use of ICIs. The severity of CAEs was classified into grades 1–5 using the Common Terminology Criteria for Adverse Events (CTCAE).

### Statistical analysis

The Fine-Gray competing risk model analysis assessed associations between baseline demographic and clinical variables and CAEs. Based on the clinical data of the patients in descriptive statistics, the results were presented as mean ± SD. Other data were evaluated for significance using the Mann-Whitney *U*-test (non-parametric), paired *t*-tests, and independent *t*-tests. The comparison data between PD-1 and PD-L1 inhibitors were processed by propensity matching with a ratio of 1:1. All statistical tests were two-sided and *p* < 0.05 was considered statistically significant. All statistics were analyzed using Stata 17 and SPSS 22.

## Results

### Predisposing factors for immune checkpoint inhibitors-related cardiovascular adverse events

**FIGURE 1:**
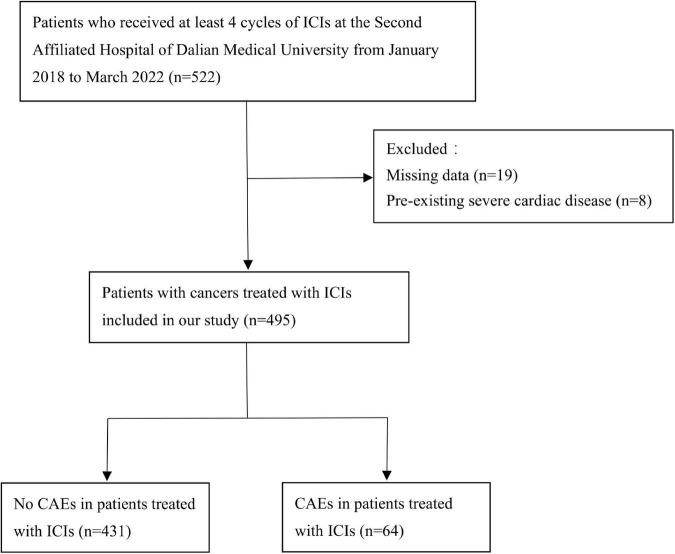
The study design for retrospective evaluation of CAEs in patients with ICIs. CAE, cardiovascular adverse events; ICI, immune check inhibitor.

After applying the inclusion and exclusion criteria, 495 patients were included in the analysis (Figure 1). Sixty-four (12.93%) patients developed CAEs within 1 year of starting treatment with ICIs. Table 1 shows the demographic and clinical characteristics of the patients. The increase in CCI score significantly increased the risk of CAEs (hazard ratio HR 1.30, 95% confidence interval CI 1.05–1.61, *P* = 0.014). A total of 218 patients had NLR < 3 (L-NLR), and 42 (19.27%) of these patients had CAEs. L-NLR was significantly associated with the risk of CAEs (HR 3.64, 95% CI 1.86–7.15, *P* = 0.000). CAEs occurred in two of ten cervical cancer patients (20.00%) after using ICIs (HR 17.61, 95% CI 1.85–167.62, *P* = 0.013). When analyzed for ICI combination therapies, VEGFR inhibitors (HR 2.57, 95% CI 1.37–4.84, *P* = 0.003) significantly increased the risk of CAEs. Twenty-four patients with a history of radiation therapy within 90 days were treated with ICIs, and three patients who received thoracic radiation therapy developed CAEs (HR 32.93, 95% CI 8.81–123.14, *P* = 0.000). Using nivolumab as a control, there were no statistically significant differences in the risk of CAEs between the eight ICIs.

**TABLE 1:** The baseline characteristics of cardiac adverse events (*n* = 495).

	No CAE (*N* = 431, 87.07%)	CAE (*N* = 64, 12.93%)	HR	95% CI	*P*
Age (years)	62.28 ± 10.18	61.78 ± 9.97	0.99	0.96–1.02	0.378
<50	42 (9.74)	8 (12.50)			
[50, 59]	104 (24.13)	14 (21.88)			
[60, 69]	201 (46.64)	29 (45.31)			
[70, 79]	67 (15.55)	13 (20.31)			
[80, 89]	17 (3.94)	0			
Sex (*n*, %)			1.97	0.96–4.05	0.065
*Male*	317 (73.55)	52 (81.25)			
*Female*	114 (26.45)	12 (18.75)			
CCI score	5.16 ± 1.88	6.03 ± 1.86	1.30	1.05–1.61	0.014
L-NLR	176 (40.84)	42 (65.63)	3.64	1.86–7.15	0.000
Tumor type (*n*, %)					
*Lung cancer*	256 (59.4)	37 (57.81)	3.80	0.71–20.41	0.119
*Stomach cancer*	32 (7.42)	6 (9.38)	4.57	0.77–27.28	0.096
*Esophageal cancer*	27 (6.26)	3(4.69)	3.41	0.45–25.99	0.237
*Liver cancer*	19 (4.41)	6 (9.38)	4.95	0.93–26.40	0.061
*Colorectal cancer*	19 (4.41)	5 (7.81)	4.42	0.95–20.53	0.058
*Cholangiocarcinoma*	6 (1.39)	1 (1.56)	6.43	0.46–90.35	0.167
*Pancreatic cancer*	6 (1.39)	1 (1.56)	4.49	0.29–69.91	0.284
*Cervical cancer*	8 (1.86)	2 (3.13)	17.61	1.85–167.62	0.013
*Lymphoma*	16 (3.71)	1 (1.56)	1.14	0.17–7.83	0.891
*Other*	45 (10.44)	5 (7.81)	4.35	0.62–30.43	0.138
Chemotherapy (*n*, %)					
*Antimetabolite*	137 (31.79)	25 (39.06)	0.75	0.32–1.76	0.503
*Anti-tubulin*	163 (37.82)	19 (29.69)	0.82	0.34–2.00	0.669
*Topoisomerase*	63 (14.62)	11 (17.19)	1.38	0.45–4.24	0.572
*Platinum*	111 (25.75)	11 (17.19)	0.73	0.36–1.48	0.382
*Alkylating agent*	5 (1.16)	1 (1.56)	2.76	0.51–14.99	0.240
Radiation therapy (*n*,%)					
*Thoracic radiotherapy*	1 (0.23)	3 (4.69)	32.93	8.81–123.14	0.000
*Radiation therapy to other sites*	17 (3.94)	3 (4.69)	0.69	0.23–2.12	0.521
Other therapies (*n*, %)					
*Anti-VEGFR*	68 (15.78)	23 (35.94)	2.57	1.37–4.84	0.003
*Anti-HER-2*	3 (0.70)	2 (3.13)	1.82	0.24–13.68	0.561
*EGFR-TKI*	59 (13.69)	15 (23.44)	1.14	0.52–2.50	0.745
ICI (n,%)					
(*ref* = *Nivolumab)*					
*Pembrolizumab*	50 (11.60)	10 (15.63)	2.43	0.47–12.64	0.292
*Atezolizumab*	17 (3.94)	2 (3.13)	0.63	0.09–4.37	0.635
*Sintilimab*	159 (36.89)	28 (43.75)	1.82	0.37–9.01	0.466
*Camrelizumab*	94 (21.81)	13 (20.31)	1.75	0.31–9.74	0.524
*Toripalimab*	17 (3.94)	1 (1.56)	0.37	0.02–5.91	0.480
*Tislelizumab*	55 (12.76)	4 (6.25)	1.21	0.18–8.04	0.843
*durvalumab*	23 (5.34)	4 (6.25)	1.30	0.17–10.14	0.802

L-NLR, low-neutrophil to lymphocyte ratio < 3; CCI, Charlson Comorbidity Index; VEGFR, vascular endothelial growth factor receptor; HER-2, human epidermal growth factor receptor 2; EGFR-TKI, epidermal growth factor receptor-tyrosine kinase inhibitor; HR, hazard ratio; CI, confidence interval.

### Classification of cardiovascular adverse events caused by immune checkpoint inhibitors

**FIGURE 2:**
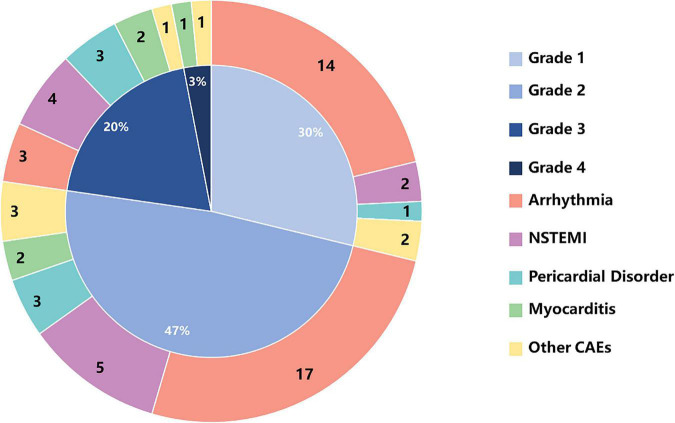
The numbers of CAEs and percentages of CAE toxicities based on CTCAE grading in cancer patients receiving ICIs. CTCAE, Common Terminology Criteria for Adverse Event; NSTEMI, non-ST-segment elevation myocardial infarction.

The most common CAE were: arrhythmia, 53.13% (34/64); acute non-ST segment elevation myocardial infarction, 17.19% (11/64); pericarditis, 10.94% (7/64); myocarditis, 7.81% (5/64); and others, 10.94% (7/64). CAE of grade 1 (30%), and grade 2 (47%) accounted for the largest proportion of total adverse events (Figure 2).

### Time of occurrence of cardiovascular adverse events

**FIGURE 3:**
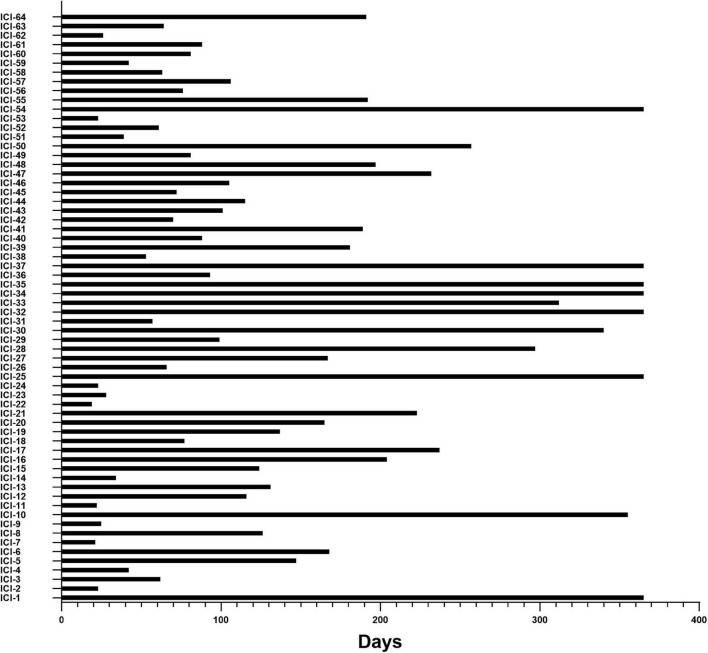
Time from initiation of ICI therapies to the occurrence of CAEs. X-axis, time (days) from cancer treatment with ICIs to the onset of CAE; Y-axis, the number of individual subjects with CAE in 64 of the 495 ICI-treated cohort patients in the study.

Among the 64 patients who developed CAE within the first year, the median time to cardiac adverse events was 105 days (interquartile range IQR: 61–202 days): 14.06% within 30 days and 68.75% within 6 months after the beginning of ICI treatment. Details are shown in Figure 3.

### Clinical and laboratory parameters of cardiovascular adverse events

Cardiac color Doppler ultrasound, electrocardiogram, and myocardial enzymes were obtained in patients with CAE. Most of the patients had normal sinus rhythm (58 ± 5%). Compared to baseline, there were significant differences in the QT interval corrected for heart rate (QTc), 449.55 ms vs. 432.79 ms (95% CI -27.02 to -6.49, *P* = 0.002), creatine kinase-MB (CK-MB), 20.29 U/L vs. 14.33 U/L (95% CI -9.73 to -2.2, *P* = 0.003), and cardiac troponin I (cTnI), 1.02 pg/mL vs. 0.46 pg/mL (95% CI -1.06 to -0.06, *P* = 0.028). The details are shown in Table 2.

**TABLE 2:** The characteristics of biomarkers in patients with CAE (*n* = 64).

Parameter, *n* (%) N		Baseline	CAEs	95% CI	*P*
WMA		0	1		
EF, %	25	59.24 ± 6.35	58.28 ± 5.25	−0.59 to −2.51	0.212
PR interval, ms	52	151.37 ± 19.18	151.73 ± 25.52	−7.77 to 7.04	0.921
QTc interval, ms	58	432.79 ± 27.06	449.55 ± 37.17	−27.02 to−6.49	0.002
CK, U/L	38	60.32 ± 31.66	394.36 ± 1148.58	−711.25 to 43.18	0.081
CK-MB, U/L	41	14.33 ± 5.72	20.29 ± 11.86	−9.73 to −2.20	0.003
cTnI, ng/ml	41	0.46 ± 0.40	1.02 ± 1.50	−1.06 to −0.06	0.028
BNP, pg/ml	12	50.95 ± 66.42	89.93 ± 118.55	−85.41 to 7.86	0.094

WMA, wall motion abnormalities; EF, ejection fraction; CK, creatine kinase; cTnI, cardiac troponin I; BNP, B-type natriuretic peptide.

### The cumulative incidence of cardiovascular adverse events of programmed cell death protein 1 and programmed death-ligand 1 inhibitors

**FIGURE 4:**
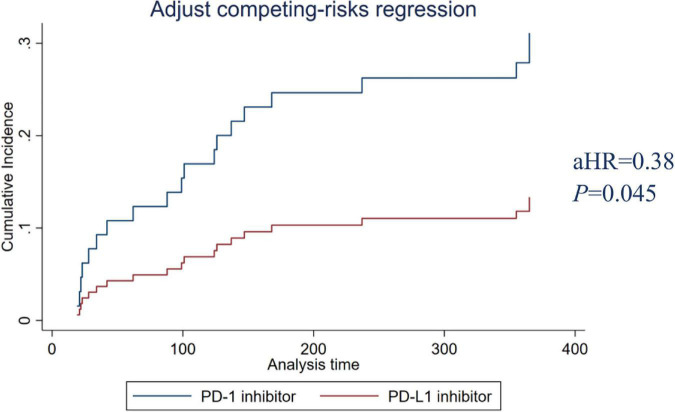
PD-1 and PD-L1 inhibitors adjusted cumulative incidence rates. PD-1, programmed cell death protein 1; PD-L1, programmed cell death-ligand 1.

Among the 495 patients analyzed, 449 (90.7%) received PD-1 inhibitors, and 46 (9.3%) received PD-L1 inhibitors. The risks of PD-1 and PD-L1 for causing CAEs were analyzed by propensity matching. Table 3 shows the characteristics of the propensity match cohort. The results of PD-1/PD-L1 before matching show that the CCI score (*P* = 0.004), L-NLR (*P* = 0.016), lung cancer (*P* = 0.000), stomach cancer (*P* = 0.04), antimetabolite (*P* = 0.003), anti-tubulin (*P* = 0.000), and topoisomerase (*P* = 0.000) had statistical differences. However, the covariates between PD-1 and PD-L1 were balanced after matching without significant differences. Results showed that PD-L1 inhibitors had a lower incidence of CAE compared with PD-1 inhibitors (adjusted hazard ratio aHR 0.38, *P* = 0.045). The adjusted cumulative incidence rates of CAE are shown in Figure 4.

**TABLE 3:** Propensity matching of patients.

Variable	Whole cohort			Propensity score matched cohort		
	PD-1 inhibitor *n* = 449	PD-L1 inhibitor *n* = 46	*P*	PD-1 inhibitor *n* = 46	PD-L1 inhibitor *n* = 46	*P*
Age (years)	61.93 ± 10.18	64.91 ± 9.50	0.058	64.17 ± 9.44	64.91 ± 9.50	0.709
<50	47 (10.47)	3 (6.52)		8 (17.39)	3 (6.52)	
[50,59]	111 (24.72)	7 (15.22)		9 (19.57)	7 (15.22)	
[60,69]	205 (45.66)	25 (54.35)		20 (43.48)	25 (54.35)	
[70,79]	71(15.81)	9 (19.56)		8 (17.39)	9 (19.56)	
[80,89]	15 (3.34)	2 (4.35)		1 (2.17)	2 (4.35)	
Sex (*n*, %)			0.336			0.615
Male	332 (73.94)	37 (80.43)		35 (76.09)	37 (80.43)	
Female	117 (26.06)	9 (19.57)		11 (23.91)	9 (19.57)	
CCI score	5.20 ± 1.91	6.02 ± 1.42	0.004	6.33 ± 1.21	6.02 ± 1.42	0.272
L-NLR	189 (42.09)	28 (60.87)	0.016	21 (45.65)	28 (60.87)	0.146
Tumor type (*n*, %)						
Lung cancer	252 (56.12)	40 (86.96)	0.000	42 (91.3)	40 (86.96)	0.505
Stomach cancer	38 (8.46)	0	0.04	1 (2.17)	0	0.317
Esophageal cancer	30 (6.68)	0	0.071	0	0	−
Liver cancer	23 (5.12)	2 (4.35)	0.819	0	2 (4.35)	0.155
Colorectal cancer	24 (5.35)	0	0.180	0	0	−
Cholangiocarcinoma	5 (1.11)	2 (4.35)	0.077	0	2 (4.35)	0.155
Pancreatic cancer	7 (1.56)	0	0.394	1 (2.17)	0	0.317
Cervical cancer	12 (2.67)	0	0.262	0	0	−
Lymphoma	17 (3.79)	0	0.180	1 (2.17)	0	0.317
Other	48 (10.69)	2 (4.35)	0.174	1 (2.17)	2 (4.35)	0.559
Chemotherapy (*n*, %)						
Antimetabolite	156 (34.74)	6 (13.04)	0.003	11 (23.91)	6 (13.04)	0.182
Anti-tubulin	176 (39.2)	6 (13.04)	0.000	8 (17.39)	6 (13.04)	0.564
Topoisomerase	44 (9.80)	30 (65.22)	0.000	27 (58.7)	30 (65.22)	0.522
Platinum	111 (24.72)	11 (23.91)	0.904	7 (15.22)	11 (23.91)	0.296
Alkylating agent	6 (1.34)	0	0.431	0	0	−
Radiation therapy (*n*, %)						
Thoracic radiotherapy	4 (0.89)	0	0.521	2 (4.35)	0	0.155
Radiation therapy to other sites	18 (4.01)	2 (4.35)	0.912	4 (8.7)	2 (4.35)	0.401
Other therapies (*n*, %)						
Anti-VEGFR	84 (18.71)	7 (15.22)	0.561	14 (30.43)	7 (15.22)	0.084
Anti-HER-2	69 (15.37)	0	0.472	0	0	−
EGFR-TKI	5 (1.11)	5 (10.87)	0.416	10 (21.74)	5 (10.87)	0.160

PD-1, programmed cell death protein 1; PD-L1, programmed cell death-ligand 1; L-NLR, low-neutrophil to lymphocyte ratio < 3; CCI, Charlson Comorbidity Index; VEGFR, vascular endothelial growth factor receptor; HER-2, human epidermal growth factor receptor 2; EGFR-TKI, epidermal growth factor receptor-tyrosine kinase inhibitor.

## Discussion

As a new method of cancer treatment, ICIs act on the immune system of cancer patients, restore the immune system’s ability to fight tumors. However, in addition to providing excellent survival benefits, ICIs can induce specific hyperactivation of immune responses, leading to non-cancer tissue damage and inevitable drug toxicity (5). Due to the rareness of cardiotoxicity, most studies present CAEs as case reports, and the incidence is often underestimated in clinical trials. Myocarditis can be more common after ICI therapies. It can develop early after initiation of ICI therapy and has a malignant course (6).

Data from a large network of healthcare organizations showed that of 5,518 cancer patients treated with at least one ICI cycle, 691 (12.5%) developed cardiotoxicity. The most common cardiotoxicity was arrhythmia (9.3%), and 2.1% of the patients developed myocarditis at 12 months (7). In this real-world study, we found that the incidence rate of CAE in patients who received at least four cycles of ICI within 1 year was 12.93%. The most common CAE was arrhythmia, accounting for 53.13% of the total CAEs. Among the 64 patients who developed CAE in the first year, the median time to diagnose CAE was 105 days, which was longer than a retrospective study of 46 days (IQR: 17–83 days) conducted from 2015 to 2018 (4). The longer time in our study could be affected by COVID-19 as it could impact hospital admissions and delay the diagnosis of patients with CAE.

One study reported that ICI-induced cardiotoxicity might be predominant in men. However, this does not necessarily represent susceptibility to men compared to women because men are overrepresented at baseline in both ICI use and clinical trial registries (8). This study showed that ICI-induced cardiotoxicity was not related to gender. Several studies have shown that CAE caused by ICI do not appear to be age-susceptible and occur in patients with a wide age range (20–90 years) (6, 9). In our study, the patients were generally older, and age had no significant effect on the occurrence of CAE. A large population-based study from multiple medical institutions showed that patients with CAEs had a higher burden of comorbidities than patients who did not develop CAEs within 1 year of ICIs initiation (*P* = 0.0042). Patients who developed CAEs were more likely to have cerebrovascular disease (12.9% vs. 10.3%, *P* = 0.0375), congestive heart failure (5.9% vs. 2.6%, *P* < 0.001), myocardial infarction (4.8% vs. 2.3%, *P* = 0.0002), peripheral vascular disease (15.6% vs. 12.3%, *P* = 0.0129), hypertension (50.8% vs. 45.1%, *P* = 0.0046), or renal disease (13.7% vs. 10.8%, *P* = 0.0221) (7). In addition, an international registry identified combination therapy, diabetes, obesity, and anti-CTLA-4 therapy were independent risk factors for cardiotoxicity (10). Pre-existing autoimmune disease may also be an independent risk factor (11). Our study also confirmed that patients who developed CAEs had a higher comorbidity burden (HR 1.30, *P* = 0.014).

NLR is the ratio of absolute neutrophil count to absolute lymphocyte count in peripheral blood, which has been shown to correlate with the prognosis of various malignancies (12). This ratio appears to reflect a balance between non-specific inflammatory and immune responses that may influence response to ICI therapies (13). Data from a retrospective analysis showed that L-NLR was significantly associated with the appearance of irAEs (OR, 2.2, *P* = 0.018) (14). Our study found that L-NLR was significantly related to the risk of CAE (HR 3.64, *P* = 0.000), which was consistent with the previous research.

With the deepening of research and the progress of clinical trials, to increase anti-tumor response, more and more ICIs are used in combination with each other or with chemotherapy, radiotherapy, and targeted therapy, increasing the risk of the complexity of toxicity (15, 16). We did not find increased risks of CAEs in patients treated with combination ICIs and chemotherapeutic drugs. VEGFR inhibitors are known to increase the risk of cardiotoxicity (17). Patients who received concomitant or previous VEGFR inhibitors combined with ICI had an increased risk of major adverse cardiovascular events (MACE) compared to patients who received ICIs alone (HR 2.15, 95% CI 1.05–4.37, *P* = 0.04) (18). The results of our study also confirmed that the combined use of VEGFR inhibitors with ICI significantly increased the risk of CAE (HR 2.57, *P* = 0.003).

The combination of radiation therapy and immunotherapy is also a hot spot in tumor treatment because radiation therapy has the effect of presenting antigens on tumor cells. The synergistic effect of radiation therapy and immunotherapy could trigger an endogenous antigen-specific immune response, thus increasing the incidence of MACE through recognizing shared antigens (19). The results of a meta-analysis showed similar grade 3–4 toxicity in ICI combined with radiation therapy (16.3%) and ICI alone (22.3%). The grade 5 toxicities were 1.1 and 1.9% for ICI alone and ICI with radiation therapy (20). A retrospective analysis found that exposure to cardiac radiation dose increased the risk of MACE. A mouse model with concurrent thoracic radiation and PD-1 blockade showed increased radiation-induced cardiotoxicity and a decreased left ventricular EF. However, there was no significant difference in cumulative chest radiation dose between the ICI and non-ICI groups (21). We found that thoracic radiation significant increased the risk of CAE (HR 32.93, *P* = 0.000). On the contrary, radiation to other sites, such as the head and neck, did not increase CAE risk.

Nivolumab was the first PD-1 antibody investigated in patients in 2006 and the first PD-1 antibody approved by the FDA in 2014 (22). On June 15, 2018, China’s Drug Administration approved nivolumab, the country’s first immuno-oncology, and the first PD-1 therapy. Since 2020, the number of pivotal clinical trials of PD1/PD-L1 drugs developed by Chinese companies has exceeded that of PD1/PD-L1 drugs developed by biopharmaceutical companies in other countries (23). In this study, there was no significant difference in CAE between domestic ICIs and imported ICIs. In addition, the effects of PD-1 and PD-L1 inhibitors on CAE were also studied. Among them, PD-1 inhibitors block the PD-1/PD-L2 pathway, resulting in increased binding of PD-L2 to the repulsive guidance molecules B (RGMb) receptor, which may affect immune system homeostasis. Anti-PD-L1 still allows PD-1 to interact with its other ligand, PD-L2, and may be less toxic as PD-L2 signaling protects immune homeostasis (24). A systematic review investigated differences in the toxicities of PD-1 and PD-L1 inhibitors in patients with non-small cell lung cancer (NSCLC). Patients treated with PD-1 inhibitors were found to have a slightly higher rate of irAEs (16% vs. 11%, *P* = 0.07) and pneumonitis (4% vs. 2%, *P* = 0.01) compared to patients who received PD-L1 inhibitors (25). Compared with PD-1 inhibitor use, PD-L1 inhibitor use was significantly associated with lower risks of cardiac complications both before and after propensity score matching (26). We found that PD-L1 inhibitors had a lower incidence of CAE than PD-1 inhibitors.

Our study found that the decrease in EF from baseline was not related to CAE, which was consistent with previous studies (6, 18), suggesting that the dependence on EF alone to detect the occurrence of CAE in patients receiving ICIs is inadequate. Clinicians should not rely on EF as a discriminant indicator of the severity of ICI-related CAE (6). The QTc interval is a standardized measure available routinely from a 12-lead ECG and predominantly represents ventricular repolarization (27). In a retrospective analysis, QTc was more prolonged (26.8 ± 12.0 from baseline; *P* = 0.036) at the time of MACE (4). Our study also showed that prolonged QTc was significantly associated with an increased risk of CAE (*P* = 0.002).

The CK value has low specificity, and our study found no significant difference in the CK value before and after the occurrence of CAE. In a retrospective analysis of patients with lung cancer treated with ICIs, mild elevations in cTnI were observed at the time of MACE (4). Abnormal levels of troponin were observed in 94% of patients with ICI-associated myocarditis (6). Elevated troponin usually indicates myocardial cell death. Our study found that elevated cTnI was significantly associated with the appearance of CAE (*P* = 0.028). Therefore, monitoring troponin in each treatment cycle could allow patients with potential myocarditis to be admitted to the hospital as soon as possible.

The strength of our study is a real-world study. Due to the low incidence of ICI-related myocarditis, pericarditis, and heart failure related to ICI, cancer patients without clinical cardiovascular symptoms treated with ICI have not received enough attention from oncologists to perform regular cardiovascular evaluations (28). Our knowledge of ICI-associated CAE has been significantly enhanced by case series and pharmacovigilance databases. Due to a retrospective design, selection bias remains a concern, as there was no prospective cardiovascular screening protocol in all sites, and screening for cardiac biomarkers and other tests was left to the discretion of the individual providers (18). In addition, due to the invasive nature of endocardial biopsy, there was a lack of biopsy-proven cases.

## Conclusion

Using nivolumab as a control, there was no independent association between the eight ICIs and CAE risk. However, PD-L1 inhibitors had a lower rate of CAE than PD-1 inhibitors. Combination therapies of ICI with VEGFR inhibitors significantly increased the risk of CAE. Patients who had a history of previous thoracic radiation therapy taking ICIs also had increased CAE risk. L-NLR and higher comorbidity burden were associated considerably with CAE and could be used as a risk predictor for CAEs. Cardiac biomarkers such as cTnI, CK-MB, and QTc were significantly elevated when CAEs were present and could be used as monitoring factors. Patients will benefit from close monitoring by incorporating clinical assessment, cardiac biomarkers, and cardiac examination into the management recommendations for ICI therapy.

## Data availability statement

The raw data supporting the conclusions of this article will be made available by the authors, without undue reservation.

## Ethics statement

The studies involving human participants were reviewed and approved by the Department of Pharmacy, The Second Affiliated Hospital of Dalian Medical University, Dalian, China. Written informed consent for participation was not required for this study in accordance with the national legislation and the institutional requirements.

## Author contributions

GF and NS: full access to all of the data in the study and take responsibility for the integrity of the data and the accuracy of the data analysis and obtained funding. SW, HB, and LZ: concept and design and drafting of the manuscript. JH, XL, and SYW: acquisition, analysis, and interpretation of data. HB, XL, and GF: critical revision of the manuscript for important intellectual content. HB and LZ: statistical analysis. SW, HB, and GF: supervision. All authors contributed to the article and approved the submitted version.
